# Opportunities for use of neuroimaging in de-risking drug development and improving clinical outcomes in psychiatry: an industry perspective

**DOI:** 10.1038/s41386-024-01970-8

**Published:** 2024-08-21

**Authors:** Amit Etkin, Jessica Powell, Adam J. Savitz

**Affiliations:** 1 Alto Neuroscience Inc., Los Altos, CA 94022 USA; 2 Department of Psychiatry and Behavioral Sciences, Stanford University, Stanford, CA 94304 USA; 3 Department of Psychiatry, Weill Cornell Medical College, New York, NY 10021 USA

**Keywords:** Predictive markers, Biomarkers, Diseases of the nervous system, Neurophysiology

## Abstract

Neuroimaging, across positron emission tomography (PET), electroencephalography (EEG), and magnetic resonance imaging (MRI), has been a mainstay of clinical neuroscience research for decades, yet has penetrated little into psychiatric drug development beyond often underpowered phase 1 studies, or into clinical care. Simultaneously, there is a pressing need to improve the probability of success in drug development, increase mechanistic diversity, and enhance clinical efficacy. These goals can be achieved by leveraging neuroimaging in a precision psychiatry framework, wherein effects of drugs on the brain are measured early in clinical development to understand dosing and indication, and then in later-stage trials to identify likely drug responders and enrich clinical trials, ultimately improving clinical outcomes. Here we examine the key variables important for success in using neuroimaging for precision psychiatry from the lens of biotechnology and pharmaceutical companies developing and deploying new drugs in psychiatry. We argue that there are clear paths for incorporating different neuroimaging modalities to de-risk subsequent development phases in the near to intermediate term, culminating in use of select neuroimaging modalities in clinical care for prescription of new precision drugs. Better outcomes through neuroimaging biomarkers, however, require a wholesale commitment to a precision psychiatry approach and will necessitate a cultural shift to align biopharma and clinical care in psychiatry to a precision orientation already routine in other areas of medicine.

## Introduction

Non-invasive imaging of the human brain has been a mainstay of clinical and translational neuroscience for over three decades. It is hard to imagine being able to understand the human brain sufficiently without being able to image its structure and function. Substantial technical and scientific advances have been made across neuroimaging modalities, yet few have entered clinical practice in psychiatry nor been systematically integrated across all stages of drug development. In the few instances where neuroimaging is used in clinical practice, it is not yet used for treatment selection. Here we outline key learnings and areas for future focus in the use of neuroimaging in both drug development and clinical practice, taking an industry perspective. Core questions addressed include how use of neuroimaging can de-risk drug development in psychiatry from target engagement through patient selection, how these biomarkers in turn impact clinical care, and what key technical, regulatory, and commercial questions need to be addressed.

Neuroimaging, defined as a set of quantitative methods for measuring the structure and/or function of the brain, includes a variety of specific techniques. This overview focuses primarily on positron emission tomography (PET), electroencephalography (EEG), and magnetic resonance imaging (MRI), as these are the most prevalent neuroimaging modalities, but the same perspectives can be applied to less frequently used or developing approaches. While some might consider EEG as a neurophysiological method distinct from MRI/PET, we do not believe this historical view is justified as EEG is in fact a more direct way to quantify neuronal function than blood flow-based measures like fMRI and at a higher temporal resolution even if it is at a lower spatial resolution. Of note, while here we focus on the development and deployment of more widely used pharmacological therapies, the same concepts can apply to other interventions, such as brain stimulation or psychosocial interventions. Overall, we argue that deep embedding of neuroimaging into both drug development and clinical care is achievable in select ways, but that to do so will require a different orientation to the collection and analyses of these data, a more tailored understanding of the utility of any given neuroimaging modality, and a cultural shift within biopharma.

**Fig. 1:**
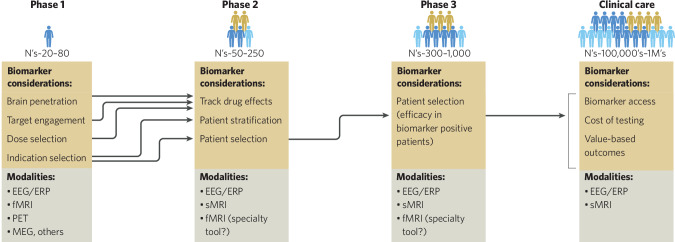
Key considerations regarding uses of neuroimaging biomarkers in Phase 1–3 of drug development as well as ultimate clinical care once a drug is approved. Elements of these biomarker uses in each phase likewise feed forward into use in the next phase, which together act to progressively de-risk development. This begins with pharmacodynamic biomarkers in Phase 1 and culminates in scalable patient selection biomarkers with drug approval and clinical deployment. Most useful modalities for each phase are noted, in line with the uses and considerations involved.

Neuroimaging has two principal contexts for use in the development and deployment of drug therapies in the near to intermediate term, with exciting additional applications in the longer-term horizon. The two current principal uses, which make up the primary focus of this piece, are as pharmacodynamic or target engagement measures to de-risk drug development and as patient stratification or selection measures to enrich clinical trials and improve clinical care outcomes (see Fig. 1). As a pharmacodynamic measure (i.e. an index of the drug’s effect on targeted brain functions), neuroimaging can be used to determine brain penetration, whether relevant brain functions are affected by a drug, the dose-response relationship for these targets, and the relationship between on-target versus off-target effects including adverse events. Pharmacodynamic measures can also be used to assess the biological impact of the intervention alongside its clinical impact, providing a link between early-stage studies and ultimate demonstration of clinical efficacy.

As a patient stratification measure, neuroimaging can be used to select patients for enrollment in a study, also generally termed an enrichment approach. For this to be successful, this use of neuroimaging would need to apply to larger scale Phase 2 and 3 trials. The anticipated result is that the drug label will consider the results of the neuroimaging biomarker in defining the on-label population, and thus the neuroimaging measure should be scalable to the millions or tens of millions of people typically impacted by any given psychiatric disorder. Longer-term uses of neuroimaging include both aspects earlier in drug development (e.g. target identification) and those more heavily grounded in clinical care (e.g. longitudinal measures guiding relapse risk determination). Each use context has additional regulatory, technical, and commercial considerations that must be addressed as the field works towards adoption into late-stage clinical development and clinical practice. We illustrate these concepts through multiple examples across drug development, as well as our own experience in doing this work (which should thus be taken as simply one example case).

## Pharmacodynamic use of neuroimaging

Clinical development plans for psychiatric medications are typically established based on an understanding of the underlying science of the drug’s molecular target and planned indication (i.e. definition of the use context within a target clinical population) and may be informed by preclinical models. Given the cost, time, and risk associated with drug development, key questions that should be answered prior to beginning clinical trials in patients are:

- Brain penetration: does the drug enter the human brain?
- Functional target engagement: what impact does the drug have on clinically relevant brain systems and functions?
- Dose selection: what is the dose-response relationship on those brain responses, and how does that inform dose selection for clinical trials?
- Indication selection: How do the brain effects drive the selection of an indication, or even a biomarker- or clinically defined subgroup within an indication?

As discussed below, different neuroimaging modalities allow various perspectives on these questions (see Fig. 1). Traditional Phase 1 studies often preclude definitive answers to these questions due to their small size and lack of statistical power, resulting in counterproductive advancement of early-stage risk into later stage trials. To mitigate this risk, we discuss important design and analytic considerations for Phase 1 trials that allow the questions above to be answered based for any specific compound.

### Molecular neuroimaging with PET

The use of PET imaging to measure occupancy by a drug of a specific molecular target relies on the ability of the administered drug to displace a radioactive tracer molecule, whose presence in particular brain regions can be detected with PET. This is one of the longest-standing forms of pharmacodynamic neuroimaging. Typically, only a small number of participants are needed to produce a sufficiently robust and generalizable Phase 1 result on PET imaging.

PET can readily answer the question of brain penetration but provides little information on the other questions. For example, it may not be known what level of target occupancy is even desired or why, and whether the dose-response relationship between target occupancy and effect on relevant brain functions is continuous or shows a threshold relationship. These relationships may furthermore differ by the specific nature of the drug’s mode of action on the target (e.g. antagonist versus inverse agonist versus negative allosteric modulator). Moreover, few PET tracers have been developed relative to the number of drug targets currently in development along with potentially druggable targets. The development of a new PET tracer is also an expensive and lengthy process with no guarantee of success, and thus is not a typical expectation for every drug development effort. Thus, while the design of Phase 1 PET studies requires little change from current practices, they are limited with respect to the degree of de-risking they provide for subsequent clinical trials.

### Functional neuroimaging with EEG or fMRI

Functional neuroimaging approaches such as resting EEG, event related potentials (ERP), resting and task-based fMRI can be used to address all of the pharmacodynamic questions above. While EEG, ERP, and fMRI-based methods each offer a different set of strengths and weaknesses (EEG/ERP with superior temporal resolution and fMRI with superior spatial resolution), the same concepts regarding design of Phase 1 efforts apply across these methods. Like PET, both EEG and fMRI can measure target engagement, but the concept they capture differs between molecular and functional neuroimaging modalities. For PET, the target being measured is at the molecular level, while for EEG and fMRI it is at the functional level. Demonstrating that a drug impacts a functional target (e.g. brain activation or connectivity outcome) provides information on brain penetration. Inclusion of multiple doses coupled with a functional outcome further allows for assessment of the dose-response or threshold relationship at this early stage in development.

The differences between molecular and functional target engagement measures can be seen in the development of drugs for cognitive impairment associated with schizophrenia (CIAS). For example, phosphodiesterase 4 inhibitors (PDE4i’s) have long been of interest as a potential pro-cognitive mechanism due to their ability to elevate the key neuroplasticity-related second messenger cyclic adenosine monophosphate [1–6]. Along with desired pro-cognitive effects, increasing doses are associated with intolerance due to adverse events like nausea, vomiting and diarrhea. The mapping between dose-response relationships across molecular, functional and tolerability measures has not been systematically described, leading to inability to successfully develop PDE4i’s in cognitive disorders such as CIAS. Recent evidence suggests that the functional (i.e. pro-cognitive) effects of PDE4i’s can be seen at sub-emetic doses [3–5, 7], which furthermore occurred at brain PDE4 target occupancy of ~30% based on translation of non-human primate PET findings [8]. We subsequently confirmed a dose-response relationship for a PDE4i on multiple cognition-related EEG/ERP signals within this range of target occupancy [6]. These results underscore the importance of doing thorough and adequately powered functional pharmacodynamic assessments. They also demonstrate a situation where PET imaging alone would have argued for use of much higher doses (and thus limited by adverse events) and missed the opportunity for pro-cognitive effects at a better tolerated dose.

Unfortunately, Phase 1 studies are presently rarely designed in a manner that uses functional neuroimaging to generate conclusions about any of the four pharmacodynamic questions. Traditional Phase 1 studies have approximately 4-6 patients per drug dose, with comparisons made across rather than within-participants. These designs are tailored for pharmacokinetics (i.e. time course for drug absorption, metabolism and elimination) and safety outcomes, but occasionally measure pharmacodynamic outcomes. Of these, EEG has been increasingly used, often due to its greater accessibility at study sites relative to fMRI. Nonetheless, because of their underpowered study designs, putative Phase 1 pharmacodynamic findings in traditional designs are heavily influenced by individual data points, variable drug levels or background biology of the participant. Indeed, if statistically significant findings are observed, these necessarily represent an unrealistically large drug effect size that is unlikely to replicate. Failure to find an effect is likewise simply a product of underpowering. Put differently, developmental risk, even around whether the drug penetrates into the human brain, is pushed to larger, slower, and more costly clinical trials, without any of the benefits of knowledge across indication or dose selection that could have been possible with a different Phase 1 approach. As many Phase 2 studies are themselves underpowered on clinical efficacy outcomes, studies may fail simply because they were set up without fundamental information on dose, pharmacodynamic effects, or indication, and not because of issues with the drug per se.

In order to de-risk later stage clinical development, the design of Phase 1 studies needs substantial change with an emphasis on adequately powering the functional outcome and a switch from a between-participant design to a better powered within-participant design. The neuroimaging modality and measures used also make a major difference. EEG/ERP tends to be highly reliable with intraclass correlation coefficients ~0.6–0.9+ [9] while resting and task-based fMRI is often ~0.4 or below [10] especially when resting-state acquisitions are <10 min long. The detectable effect size is the true effect size multiplied by the square root of reliability [11], meaning that a true biological effect size would be substantially harder to detect with fMRI relative to EEG/ERP in their current forms, necessitating larger effect sizes or much longer scan times. Not included in these reliability estimates is the additional advantage of easier standardization of EEG/ERP acquisition across multiple study sites by deploying identical hardware and operator training. Greater variance across these procedural considerations will only serve to further weaken reliability. For these reasons, in our recent EEG dose-response study of the pharmacodynamic effects of a PDE4i, we conducted a 40-person within-participant cross-over design study, giving us more statistical power to detect medium effect size outcomes [6]. Furthermore, in our own experience, we have found that employing custom software tools with near real-time quality control results in consistently high rates of satisfactory data even when collected by study staff at multiple sites with little to no prior EEG/ERP experience, as exemplified by two large biomarker-driven Phase 2b trials each of which involves over 30 sites (https://Clinicaltrials.Gov/Study/NCT05712187, https://Clinicaltrials.Gov/Study/NCT05922878).

Another facet that can be improved is the breadth of indication-relevant pharmacodynamic tests, especially when framed as an opportunity to identify unanticipated target indications. Most often, resting EEG has been used though its utility has at times been limited by experimentors not collecting both eyes open and eyes closed conditions, or using inadequate acquisition durations. ERP measures may also be collected, but attention is needed to the breadth of those measures. For example, as ERP measures relevant to CIAS (e.g. mismatch negativity) are fairly well defined in the literature [12–14], a Phase 1 study may include several of these measures, enabling detection of putative pro-cognitive effects. However, if the drug has effects on other facets of cognition, emotional processing, reward responsivity, or decision-making, not collecting this type of information means that a study serves as confirmatory for a narrow indication set but is not informative regarding other indications (e.g. mood, anxiety, etc). This can be addressed in several ways including indexing drug-induced EEG changes against EEG data collected on patients with a variety of clinical indications, as well as by leveraging additional experimental paradigms developed across the cognitive and affective clinical neuroscience literature. In some cases, such paradigms may have already been sufficiently validated to be employed with minimal change; while for others, additional validation and optimization work may be required for repeated measurement of within-participant intervention effects.

In summary, addressing all four of the pharmacodynamic questions is feasible but is still rarely done in drug development. More broadly, there is no fundamental reason why functional neuroimaging should not be positioned as a key gating step in decisions on whether and how to advance a drug into clinical trials, since doing so will directly de-risk subsequent development phases. Likewise, the current state of neuroimaging technologies is not an inherent limitation for broader use of decision-supportive Phase 1 pharmacodynamic studies, wherein issues like study size and design are more often the limiting factor.

### Future directions

**Fig. 2:**
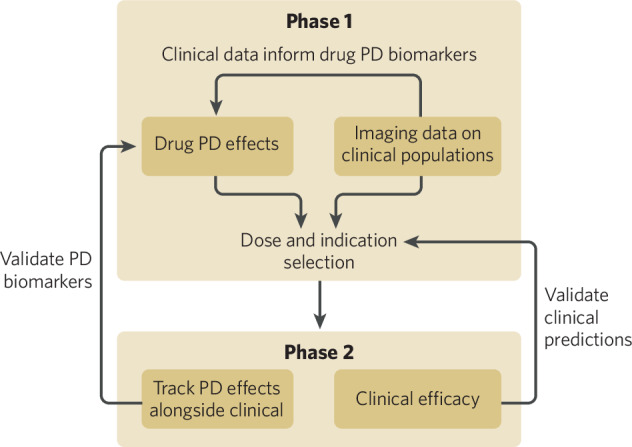
Feed-forward and feed-back flow of biomarker-related information on pharmacodynamic and clinical effects by systematically collecting biomarkers across drugs, populations and development phases. Through iterative cycling through these processes over time and development programs, the predictive utility of early biomarker outcomes on later clinical development risk and clinical efficacy can be tightened, approximating progress already being made in other areas of biomedicine.

Beyond greater emphasis on pharmacodynamic measures in Phase 1 studies, future work should focus on strengthening the relationship between demonstrating effects on these measures and subsequent clinical efficacy of the drug (see Fig. 2). In other areas of medicine, a change in a biomarker is even enough to support accelerated approval given its relationship with core disease processes or as a proxy for ultimate clinical outcome. One such example is plasma neurofilament light chain levels, which indicate levels of neuronal damage and thus neuroprotective effects of a drug. The effect of tofersen on neurofilament levels drove its recent accelerated approval for a specific form of amyotrophic lateral sclerosis, with the expectation that neurofilament reduction will ultimately lead to clinical benefit (https://Www.Fda.Gov/Drugs/News-Events-Human-Drugs/Fda-Approves-Treatment-Amyotrophic-Lateral-Sclerosis-Associated-Mutation-Sod1-Gene). Psychiatry, however, presently suffers from two limitations with respect to functional neuroimaging biomarkers in this vein. One is the relative paucity of well-validated measures, which as noted above are richer for CIAS and cognition than other areas of psychiatric illness. The second is the general lack of a well-understood relationship between how a drug impacts a specific neuroimaging measure and the likelihood of that drug having clinical efficacy. For example, though mismatch negativity has been extensively validated as a measure of early stimulus processing abnormalities in schizophrenia, a drug being able to improve this measure has not yet been shown to have clinical efficacy [15]. Mismatch negativity is also one of multiple candidate CIAS-relevant biomarkers; systematic examination of these across clinical populations, Phase 1 pharmacodynamic effects [6] and ultimate clinically-relevant changes may reveal that EEG/ERP biomarkers other than mismatch negativity are better suited for this purpose. The ability furthermore to assess biomarkers that can be similarly measured in animals enables a more mechanistic understanding of a drug’s effect on these signals, which can in turn further improve the ability to target the right clinical populations.

Efforts to standardize and validate pharmacodynamic measures have slowly evolved, such as through the work of the pre-competitive ERP Biomarker Qualification Consortium [14]. Similarly, the Autism Biomarkers Consortium for Clinical Trials (ABC-CT) has submitted the first candidate psychiatric biomarker through the FDA’s drug development tools program, making use of the latency of the N170 face-response ERP for the purpose of patient stratification/enrichment (https://Www.Fda.Gov/Media/127495/Download) [16, 17]. Much more work is needed, especially in areas such as depressive disorders where neurobiological conceptualization of core features such as low mood remain vague. However, substantial nearer term progress can be made simply by systematically carrying forward Phase 1 pharmacodynamic measures into Phase 2 and 3 studies. There are several ways in which doing so carries tremendous potential.

First, though functional neuroimaging in Phase 1 can address the pharmacodynamic questions posed, the degree to which these answers are predictive of later clinical success varies. Closing the loop between Phase 1 biomarker effects and Phase 2/3 clinical efficacy requires measuring the same pharmacodynamic signals in later stage trials (Figs. 1 and 2). Standardizing this approach over multiple clinical development programs will indicate which pharmacodynamic effects are most predictive for which sorts of clinical outcomes and which may be very helpful for addressing some early-stage questions but are not directly predictive of clinical efficacy. Without knowing the pharmacodynamic effects in later stage clinical trials, one does not know if the failure on clinical measures is due to the lack of translatability of the pharmacodynamic marker or a failure to have an effect on the pharmacodynamic marker in a larger clinical trial population. Cognition-relevant neuroimaging biomarkers may be the first to see success in this regard, as cognitive change itself can support approval in areas such as CIAS. In a related vein, the study of the effect of kappa opioid receptor antagonists on reward processing (as a pharmacodynamic measure, measured with fMRI) has motivated its development for depression patients with prominent anhedonia symptoms [18]. A subsequent clinical trial suggests that those patients may respond better than depression patients with lower levels of anhedonia [19].

The additional benefit of carrying pharmacodynamic measures into Phase 2/3 studies is that changes in neuroimaging biomarkers over longer time frames than possible in Phase 1 studies can be examined alongside clinical impact. This may even enable patient-level tracking of the biological effect of the drug to aid interpretation of its clinical effect and inform the temporal sequence of drug effects. Given its flexibility and scalability, EEG/ERP is likely the modality best suited for creating the bridge between early and mid/late-stage trials as it can be measured in the hundreds or thousands of patients that will go through a clinical development program.

A second intriguing possibility is a “digital twin” approach that uses machine learning to decode various dimensions of drug effect on Phase 1 neuroimaging data which then allows one to run in silico “virtual trials” by leveraging data across various clinical indications to mine for a specific neural signature (see Fig. 2). The digital twin concept originated with the NASA Apollo mission but was formalized in the context of manufacturing, and has more recently been taken into healthcare [20–23]. A digital twin is meant to be a model with a bidirectional relationship with its real-world counterpart – informed by observed data and in turn driving hypotheses and interventions. The challenge with use of digital twins in healthcare compared to manufacturing is that the latter is engineered whereas in healthcare, a working mechanistic model must be iteratively discovered and refined through experimentation, an area where neuroscience lags other biomedical fields. Examples of successful digital twin approaches in healthcare include the VICTRE study (the Virtual Imaging Clinical Trial for Regulatory Evaluation) that evaluated various imaging technologies in the detection of breast lesions, as well as use of synthetic control arms to expand the coverage or scope for several oncology drugs [24].

In the present context, in silico or virtual trials can be thought of in two complementary ways. In one, the neural signature(s) of a drug (relative to placebo) are determined, and then data from patients are searched to find those indications or subgroups that best match this pattern or its inverse. In a complementary approach, a library of signatures of clinical groups is determined and then the Phase 1 data are assessed for discrimination between drug and placebo based on a pre-determined clinical signature. In either case, the goal is to match a potentially rich set of neural changes between drug effect and a variety of clinical populations in order to drive evidence-based indication or subgroup selection. Here, a resting scan (EEG or fMRI) may be optimal as it can capture changes unconstrained by a task-based context and capture the multiple potentially independent dimensions of brain function relevant to drug or disease, building upon historical “lock and key” concepts [25]. A similar approach is already well established using genetic or genomic data to find opportunities for drug repurposing or targeting, where it is a well-accepted analytic strategy [26–29]. An example of the digital twin gene expression framework for drug discovery can also be found in the domain of immunology and inflammation [30, 31]. A digital twin approach could also have the possibility of revealing surprising hypotheses regarding unanticipated indications, accelerate identification of candidate indication subgroups, allow for a better understanding of drug mechanisms across multiple measures and/or modalities, and may help inform novel mechanisms of action of drugs undergoing first-in-human testing. Nonetheless, one of the biggest challenges for developing digital twins in psychiatry is the relative paucity of available neuroimaging data, as well as the early stage of this work in healthcare overall based on the need for experimentally determined vs engineered digital twins. The degree to which there will be future regulatory acceptance in psychiatry for registrational studies of digital twin approaches (e.g. synthetic control arms) is presently unknown, meaning that the greatest utility of such concepts may be in the domains of indication and subgroup selection.

Though hard to presently imagine given the current state of psychiatric drug development, it may be possible in the future through systematic collection of pharmacodynamic data across phases of development to arrive at the point where a Phase 1 result is reasonably predictive of clinical efficacy in larger pivotal trials. That was, for example, the hope of early work on the mismatch negativity with respect to utility in schizophrenia [15]. This is already frequently the case in other neurological disease areas (e.g. amyotrophic lateral sclerosis, Alzheimer’s disease), in the examples of neurofilament light chain or beta amyloid levels. The success of this type of approach in psychiatry is primarily limited by the lack of systematic and appropriately designed data collection efforts rather than a fundamental limitation in our understanding of psychiatric disorders or the brain more generally. Put differently, a pharmacodynamic measure may prove consistently predictive of clinical utility even in absence of fully understanding the underlying biology.

## Use of neuroimaging for patient stratification and selection

The goal of precision psychiatry is to use biomarkers, such as those derived from neuroimaging, to identify the patients best suited for a particular treatment. In practical terms this entails both patient stratification and selection in Phase 2/3 trials as well as deployment of the neuroimaging biomarker at scale in clinical practice since the on-label population will likely require the test be performed (see regulatory considerations section below; see Fig. 1). Patient stratification and selection de-risks clinical development by identifying patient subpopulations for which larger effects of the drug (relative to placebo) are expected. It also improves ultimate clinical outcomes if the raw response rate to the drug is itself magnified in biomarker-defined patient subgroups. Furthermore, by increasing clinical efficacy through an objective and systematic approach at patient identification, the scaling of clinical development from smaller Phase 2 studies to large-scale Phase 3 studies is less likely to result in effect size degradation, as is commonly seen. Hence the following are key questions any potential neuroimaging stratification signal must address:

- Reliability: is the biomarker stable over a long enough period to support prescribing decisions?
- Robustness and reproducibility: is the ability to predict better outcome consistent across different clinical populations and treatment contexts?
- Scalability: can a measure used in R&D scale to the millions of people with the target indication(s)?
- Cost, accessibility, and role in the patient journey: at what point in the patient journey is testing for the biomarker anticipated, will it be done once or multiple times, and how does the cost of doing so impact the ability of patients to get the test and associated treatment?

### Impact of commercial and clinical feasibility on design of clinical trials

Unlike in academia, where the outcome of a clinical trial is principally the knowledge it generates, in industry the primary goal of research efforts is to produce a drug that will enter clinical care. Thus, consideration of this goal in the design and execution of clinical trials in Phase 2 and 3 is critical to the ultimate clinical utility of the drug. An important set of factors that often differ between clinical trials and clinical care are the diversity of patients assessed and treated (clinically, biologically, concomitant medication use, etc) and the time between when a biomarker test is performed and drug treatment initiated. As such, reliability and robustness of a neuroimaging modality is essential to its utility. For example, given the low feature-level reliability of conventional fMRI relative to both EEG/ERP (see above) and structural MRI (intraclass correlation ~0.9+), fMRI in its present form is unlikely to be useful as a clinical biomarker unless substantial advances are made in its collection. Multivariate methods might improve reliability of fMRI and improve its sensitivity [32, 33], though fMRI research must still address the harmonization across scanners and extension to non-academic imaging facilities, thus requiring substantial future methodological development. Reliability also increases with very long scan times [34], increasing the importance of addressing challenges around cost efficiency and scalability of fMRI biomarkers. Neuroimaging biomarkers collected during development may also be less useful in clinical care if the biomarker is seen in only a small portion of the total illness population or is tested on an overly restricted population definition that is not representative of patients seen in regular clinical care.

However, reliability and robustness are by themselves not sufficient. The amount of clinically relevant information captured by any given neuroimaging modality is also important. For example, a recent systematic review found that the most robust and prospectively replicated treatment response prediction signals in depression come from EEG, with weak replication and small effects in fMRI, and no replication in sMRI [35]. EEG signals such as individual alpha peak frequency, an index of vigilance, and machine-learning derived multivariate patterns have been independently replicated as predictors of treatment outcome in depression [36–40]. fMRI, despite its lower reliability, has even outperformed structural MRI from a predictive perspective [33]. fMRI’s ability to capture deep brain activity and at a higher spatial resolution may support its predictive utility in certain contexts. One challenge, however, to subcortical fMRI signal utility is the lower test-retest reliability in these regions [41, 42].

None of these findings necessarily reflect an invariant aspect of psychiatric neuroimaging and may also differ in application between disorders featuring a slow time-course of progressive change (e.g. neurodegeneration) as compared to episodic conditions (e.g. mood disorders). Nonetheless, they together should inform what types of metrics are collected in any given development program, with further emphasis placed on the consistency with which any individual modality is captured across studies in order to generate sufficient data.

Another factor is the real-world cost and scalability of a neuroimaging modality and balancing this against the value it delivers. Though cost and scalability will determine utility in the context of similar reliability, robustness, and information content, these practical factors can quickly outweigh the scientific ones. Consider for example the use of amyloid PET imaging in Alzheimer’s disease. Verification of the presence of amyloid beta pathology is required in the label for lecanemab (https://Www.Accessdata.Fda.Gov/Drugsatfda_docs/Label/2023/761269Orig1s001lbl.Pdf), which is conventionally done with PET imaging. This is thus an example of a biomarker-based enrichment design for clinical trials, carried subsequently into clinical care. However, despite the very high reliability and extensive research experience of amyloid PET [43], it remains costly and not universally available. Already there are FDA-cleared cerebrospinal fluid tests for beta-amyloid with results equivalent to PET (https://Www.Accessdata.Fda.Gov/Cdrh_docs/Pdf22/K221842.Pdf) [44], which may provide advantages on both cost and scalability relative to PET, and in the future potentially blood tests as well [45]. Likewise, future enrichment-based clinical trial designs targeting amyloid neuropathology may themselves switch to using blood or cerebrospinal fluid amyloid levels rather than PET measures.

While PET is unlikely to be a biomarker in psychiatry based on the status of current research, the example above raises questions about considerations between EEG/ERP and fMRI. The per-assessment cost of fMRI is likely to remain substantially higher than for EEG/ERP. The further potential that assessments would need to be done at multiple points over a treatment course would only exacerbate this pragmatic difference. These real-world cost considerations will likely impact when in the patient journey which kind of biomarker is used. For example, lower cost and easy to repeat neuroimaging modalities may be used in more people and beginning earlier in the course of treatment. By contrast, more expensive and specialized technologies such as MRI may find a home in later-stage and more costly portions of the patient journey, where spatial resolution or imaging of subcortical structures are important, such as in guiding invasive neurostimulation treatments wherein spatial precision matters more. EEG systems have themselves rapidly advanced over recent years with technology such as dry electrodes, ambulatory at-home uses, as well as existence of a number of low-cost consumer-grade devices. Thus, it is reasonable to expect that home-based EEG assessments can be done routinely at scale with device manufacturing costs <$1,000 in the near term. This cost point, coupled with neuroimaging that can be self-administered by the patient through guidance by tailored acquisition software, additionally enables the potential for repeated or longitudinal assessments (see section below) which itself opens new horizons for biomarker utility in psychiatry.

As such it is an open question whether investment in fMRI as part of Phase 2/3 trials is worth the cost and challenges of ultimate clinical-scale implementation, or rather whether the field should focus on EEG/ERP data, for which technology already supports its systematic collection (even if not currently routine in trials; see Fig. 1). For fMRI to be useful, its superior spatial resolution and ability to directly image subcortical structures must be shown. For example, it is assumed that fMRI’s ability to capture subcortical activity is important, but only very rarely is the effect of removing these regions from an fMRI classifier tested. If doing so does not degrade performance (e.g. because the bulk of brain signal by total volume is cortical), this would cast doubt on this assumption. Indeed, the rise of surface-based analytic methods has led many researchers to only use cortical signal. This concern similarly holds for demonstrating utility of the spatial resolution advantage of fMRI, especially in the context of a biomarker for drug treatment. It is possible that fMRI’s spatial resolution may be important for guiding neurostimulation treatments, though thus far fMRI guidance of TMS only explains approximately 3% of variance in outcome [46] and no benefit has yet been found for neuronavigation of TMS compared to crude scalp positioning [47].

### Prospective replication is critical to biomarker development

The history of drug development is full of examples of post-hoc data analyses conducted after a trial fails, wherein the developer suggests that using a different trial design or patient subgroup may yield a positive outcome. These analyses are typically either unconstrained or constrained to only a limited degree with regard to how many comparisons are being made (with no correction for multiplicity). As a consequence, subsequent trials based on these findings rarely yield positive results. It is also likewise well-documented that the kind of machine learning analyses increasingly used to develop candidate biomarker signals can overfit on the data on which they are trained and not generalize to other samples [48, 49]. Nonetheless, analysis of patient data is often critical to finding a more biologically homogenous patient population that is more likely to respond given the high degree of biological heterogeneity embedded within current phenomenologically-defined diagnoses in psychiatry.

**Fig. 3:**
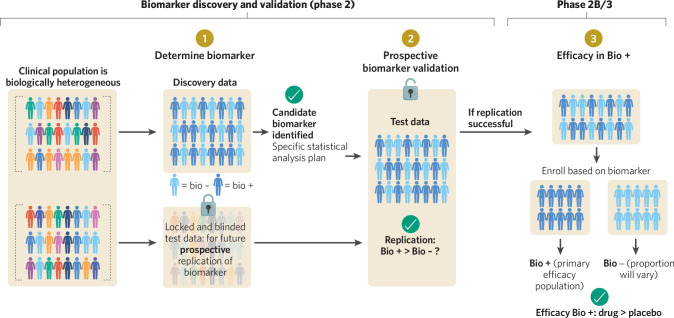
Conceptual overview of the development and validation of a biomarker-based patient selection approach in clinical trials. Central to this approach is the requirement for prospective validation of a biomarker’s ability to stratify populations on clinical outcome in an independent group of patients. Upon successful replication, a patient selection approach can be taken, in line with the FDA’s enrichment guidelines.

It is thus critical that any candidate biomarker being considered for use in a patient stratification and selection approach be prospectively replicated in a completely independent patient cohort not used in any part of the analyses to determine the biomarker (see Fig. 3). This is different from the cross-validation approach often used in neuroimaging machine learning analyses and is more costly with regard to the amount of data (i.e. number of participants) needed to be collected in trials. This approach, and pre-specification of analyses more broadly, is still infrequently used in psychiatric academic neuroscience, often because too little data are available in studies, or the neuroimaging modality is so highly dimensional that more data needs to be used in training to better fit the outcome being predicted. In drug development, neuroimaging data are rarely collected in Phase 2 studies and even less frequently in Phase 3 studies, leading to even less available data. However, given the expectation in industry that analysis of clinical trial data be directed through pre-specified statistical analyses plans, use of independent data to prospectively test the ability of a biomarker to predict outcome can be conceptualized under a similar framework. In our own work, we have required that candidate patient selection biomarkers be prospectively replicated in an independent cohort of patients receiving the study drug. This was already successful in two programs in depression [50, 51]. Both of these have advanced to Phase 2b trials wherein patients are selected based on a combination of their diagnosis (major depressive disorder) and status on a biomarker test comprised of either performance in a test of memory or a specific EEG pattern (https://Clinicaltrials.Gov/Study/NCT05712187, https://Clinicaltrials.Gov/Study/NCT05922878).

**Fig. 4:**
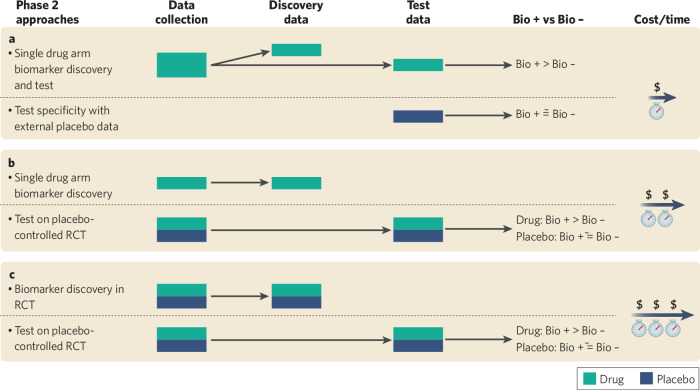
Different Phase 2 trial designs for the development and validation of a biomarker for patient selection. Three example approaches of progressively greater cost and time requirements are shown, along with how discovery and test data are divided for analysis and associated predictions. The first approach (**a**) is best suited for situations where external placebo datasets exists in which relevant biomarkers were collected. The use of a single drug arm design is the most streamlined way to identify and replicate a patient selection biomarker that will also translate best to how the drug would be used in clinical practice. The second and third approaches (**b**, **c**) are best suited to indications in which little data are available and biomarker-related knowledge must be generated entirely through the data being collected (RCT = randomized clinical trial). If there is substantial concern about whether biomarker discovery depends on drug exposure context (single arm vs. RCT) then the third approach (**c**) may be best. However, in this situation, there may be concern about how well a biomarker will generalize to clinical care where treatment more closely resembles the single arm context.

Examples of Phase 2 trial designs to accomplish the goals of biomarker discovery and prospective replication in independent data are shown in Fig. 4. Selection between these various options is driven by a variety of considerations, including whether biomarker data are already available on placebo-treated patients, relationship between biomarker use in randomized trials versus clinical care, time, and cost. Regardless of the approach taken, for any biomarker to advance to Phase 2b/3 studies for use in patient selection it must both predict better drug response in the replication data as well as not enrich for better placebo response. While there may also be utility for a biomarker that predicts better response to a drug relative to placebo without enriching for greater absolute drug response (i.e. because it predicts lower placebo response), the clinical utility of this biomarker profile is unclear. It may be the case that patients characterized by this biomarker have a lower base rate of response naturalistically, as reflected in a trial by lower placebo response, and that the drug is therefore effective in these patients despite greater background treatment resistance. However, how this would be viewed from a regulatory perspective with respect to who the on-label population would be is unknown. The more conventional expectation of a precision psychiatry approach is that if a biomarker is used for patient selection and key to the drug label, it should be associated with an overall better response to the drug in those positive for the biomarker.

### Drug development success requires a commitment to a precision approach

Structural and conceptual challenges also exist for incorporation of neuroimaging-based stratification in drug development. Integration of neuroimaging requires sufficient technical and analytical expertise in the relevant modality. Sponsor companies may not have developed internal expertise and few CROs have adequate expertise likely due to a historical lack of inclusion of such data. We strongly recommend building in-house teams and processes. Doing so will control costs, improve standardization across sites, and provide for adequate internal oversight of data. Investing in these resources early in development will also improve the ability to translate neuroimaging findings to clinical practice upon commercialization. We have done so in our own work, building an internal clinical operations team (which works directly with study sites), engineering team (which develops biomarker assessment methods and software) and data science team (to identify biomarkers), all fit-for-purpose for a drug development approach in which pharmacodynamic and patient selection biomarkers are central.

More generally, despite patient selection being expected in areas such as oncology [52, 53] (e.g. testing for mutations such as those in EGFR which lead to sensitivity to gefitinib in non-small cell lung cancer), this perspective is still rare in psychiatry within either clinical development or commercial portions of drug development organizations. Explicit in oncology is that precision leads to larger effect sizes, greater predictability of late-stage trials from early-stage results, and an overall higher probability of success with often smaller sample sizes. Indeed, the trend in oncology is toward progressively greater population fractionation in the search for ever more effective treatments [54], as a consequence of which there are far more oncology drugs in development than for any other area [55]. This has resulted in a 70% rise in oncology drug revenue for pharma companies between 2010-2019, as contrasted with the 18% reduction in non-oncology revenue [56]. Thus, a deep commitment to precision drug development in oncology has benefitted both clinical outcomes for patients as well as revenue for drug developers.

By contrast, the lack of a systematic commitment to a precision psychiatry approach leads to under-investment in building the teams and tools required, further exacerbating the challenges of collecting data that validates a biomarker-based patient selection approach. Successful collection and stratification using neuroimaging requires development of custom software tools, integration of software into the requirements of specific hardware, and establishment of methods for near real-time quality control and biomarker assessment. Real-time assessment is particularly critical when prospectively enrolling participants based on a neuroimaging signal. This is not about simply collecting more data in trials, but a wholesale shift in expectations for the design of trials and consequently the deployment of biomarkers in clinical practice at the same time as not overly increasing the site or participant burden in clinical trials. Successful implementation requires a substantial commitment of time and money from knowledgeable groups and thus requires dedicated effort and attention.

In sum, what is needed is a full commitment to a precision development path, with all of the attendant investment in people and resources. This requires a cultural shift in biotech/pharma and repeated demonstration that this approach yields better outcomes and higher probabilities of developmental success. While it is hard to imagine a future in psychiatry without precision, given both historically high psychiatric failure rates and the successes of precision medicine in areas like oncology and immunology, such a cultural shift in psychiatry may take time for many organizations. Nonetheless, the resources that biopharma can marshal against a challenge once a potential solution presents itself raises hope that when this cultural shift happens, outcomes will follow. Examples of early evidence of both the deployment of such resources and acknowledgement of the unique commercial value of a precision approach can be seen in the Phase 3 development of two antidepressants by Janssen Pharmaceuticals – seltorexant and aticaprant. Seltorexant has demonstrated a larger response in patients with elevated insomnia symptoms [57] while aticaprant has shown a larger response in more anhedonic patients [19]. Though neither make use of neuroimaging-based patient selection biomarker, they nonetheless capitalize on the FDA’s enrichment guidelines (see below) and reflect an important step towards broader biology-defined precision psychiatry.

## Regulatory, clinical adoption and commercial considerations

While no drug has yet been approved in psychiatry explicitly through a precision development approach, there are multiple lines of evidence pointing to a path forward from a regulatory perspective. Zuranolone, for example, was only approved for a subset of patients with depression with postpartum onset, but not for major depression more broadly. This accords with effect sizes for this drug, which have been substantially larger in postpartum depression than in a broader unselected depression population [58–60], as well as biological rationale around postpartum changes in neurosteroid levels. While zuranolone is probably the clearest case amongst approved drugs for a precision approach, drug approvals for treatment resistant depression have also relied on arbitrary definitions of that subgroup relative to depression as a whole (albeit they have not generally shown preferential responsivity in that subgroup).

The FDA has also published general guidance around enrichment-based designs, which can be employed in designing psychiatric trials in which neuroimaging biomarkers are used for patient selection (https://Www.Fda.Gov/Media/121320/Download). While suggested designs differ, they generally focus analyses on patients positive for the biomarker (enrichment marker), which need to be well-enough defined and powered as the primary efficacy population. Patients without the biomarker are also often included, though the degree to which this population is represented relative to the biomarker positive sample may differ across designs depending on risk-benefit and biological rationale considerations (see Fig. 3). The guidance also notes that the use of an enrichment design will have relevance to drug labeling.

An additional factor to consider is that a neuroimaging patient selection marker will likely also require a parallel clearance as software as a medical device (for scoring the biomarker), along with a potential clearance of the neuroimaging device itself if not already cleared. Thus, a neuroimaging-based enrichment-driven drug development effort must include engaging with both the drug and device divisions within the FDA. These factors may increase cost and complexity required for precision drug development.

Beyond approval, clinical adoption will require education of clinicians on the background and rationale for the biomarker as part of the drug launch. As such, making the biomarker easy to collect and interpret will also be important. If an EEG biomarker test can be performed by the patient on themselves in their home (which is feasible with current or near-term EEG technology), then anybody can acquire the same data in any clinical context. By contrast, if the patient needs to be sent to a radiology center to get an fMRI scan, this may limit the accessibility and adoption of the biomarker and associated drug. The easier and more scalable a biomarker is, the more likely it will be able to also engage patients themselves in changing clinical practice given the widespread frustration of patients with the trial-and-error status quo. Further, the clearer and simpler the results (i.e. positive/negative on a biomarker test) the faster adoption will be by reducing requirements for expertise, which at this point remains rare in psychiatry.

Likewise, whether and how much separate insurance reimbursement is sought for the biomarker assessment itself will determine the way in which the drug and biomarker are used in the clinic. To which end, also relevant is the business model of the drug developer itself, which will depend on whether the revenue streams for the biomarker and drug are through different entities (as often the case in oncology, requiring separate revenue drivers) or if they are both controlled by the drug developer (which can consider together the overall drug plus biomarker revenue). Moreover, if a biomarker can be administered at low cost (e.g. via a home EEG test) and is seen principally as a way to identify candidates for a drug being developed by the same entity, a need for reimbursement for the biomarker itself may not even be required for the overall drug plus biomarker business model to succeed. By contrast, if the biomarker is itself costly (e.g. fMRI, especially if requiring multiple or lengthy scans), this may add further cost and adoption challenges to its use prior to a patient being identified as a likely drug responder and contributing to this likely larger source of revenue. For such costly biomarkers, an additional challenge is the adoption of Current Procedural Terminology (CPT) codes and medicare reimbursement, such as happened for beta amyloid PET imaging.

We illustrate these practical considerations from the perspective of our own experience across two different biomarker-driven drug development programs in depression. The patient selection biomarker for ALTO-100 is a memory test [50] implemented in a web-based self-administered behavioral battery. As such, delivering scalable access to this test is readily accomplishable with very limited cost. For ALTO-300, the biomarker is EEG-based [51], but done using a lower electrode count montage (i.e. not the 64+ channels typical in academic research environments) based on a single session of a brief resting-state recording, with a substantial proportion of EEGs already being collected in patients’ homes in our trials. Solving for how to deliver this test at low cost thus becomes a technical and logistical process around use of simpler EEG systems. Importantly, by fully committing to a precision psychiatry development strategy, driving both drug and biomarker development under a single roof, and embedding both within a single business model, we anticipate having the flexibility needed to enable entry of these precision therapeutics and their attendant biomarkers into clinical care, pending outcomes from our trials.

## New frontiers in use of neuroimaging

In particular because of availability of low-cost, easy to use EEG devices, the potential exists for transition from a one-time treatment selection approach to one where longitudinal monitoring of brain changes can inform clinical care. In the context of drug treatment, this could take the form of prediction of risk for another episode, monitoring of drug effects beyond those evident in clinical changes, or even guiding the dosing regimen itself. On this topic, the science remains nascent, but it is likely that as neuroimaging biomarkers become used with patient selection, researchers and companies will begin to look for new ways to deploy these tools throughout the patient journey.

As neuroimaging data amass at a clinical scale after approval of the first drug-biomarker combinations (as a contrast to the scale possible in research alone), another potential use exists for identifying novel molecular targets. By using neuroimaging to define specific biological phenotypes, molecular correlates can be sought through well-powered genetic association studies. Gathering genetic data when hundreds of thousands or millions of people are undergoing biomarker testing may require only an incrementally broader infrastructure but yield new horizons in terms of future drug targets. Likewise, initial work has also found that understanding different spatial patterns of brain functional changes (e.g. related to diseases or patient subtypes) may inform target discovery through correlation with spatial transcriptomic information [61, 62]. Thus far, this has been more limited by lack of large and robust sources of spatial transcriptomic information on human brains but is nonetheless plausible as the field progresses.

## Conclusion

Here we have taken an industry lens to examine the opportunities and challenges in using neuroimaging to both de-risk drug development and to improve clinical efficacy in psychiatric practice. Despite the limited degree to which neuroimaging is presently used in drug development, with almost no use in clinical care, paths are evident for incorporation of different neuroimaging modalities over the near to intermediate term. Better outcomes through neuroimaging biomarkers, however, requires a commitment to a taking a precision psychiatry approach and will necessitate a cultural shift to align biopharma in psychiatry with the precision orientation already routine in other areas of medicine.
